# Supplementary material for: Priming with LSD1 inhibitors promotes the persistence and antitumor effect of adoptively transferred T cells
Source: Nat Commun. 2024 May 21;15:4327. doi: 10.1038/s41467-024-48607-4 (PMC11109160; doi:10.1038/s41467-024-48607-4)
Supplement: Supplementary file 1 — Supplementary Information [file 41467_2024_48607_MOESM1_ESM.pdf]

Supplementary Figure 1

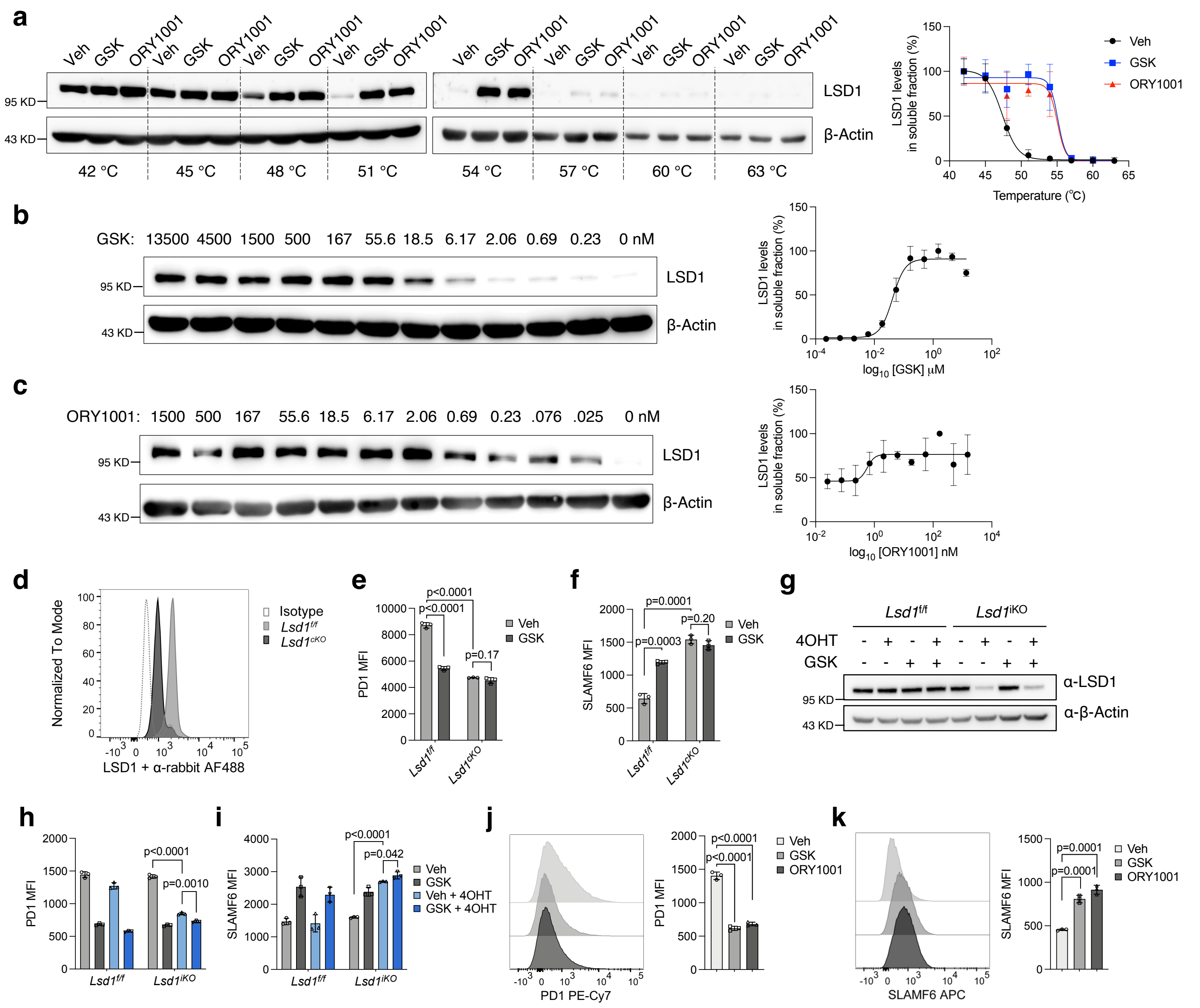

Supplementary Fig. 1 GSK and ORY1001 target LSD1 to regulate CD8⁺ T cells.

a CETSA melting curves of LSD1 in activated $CD8^+$ T cells treated with 0.5 µM GSK, 0.5 µM ORY1001, or Veh for 1 h (n=3).

b, c CETSA dose response curves of LSD1 in activated $CD8^+$ T cells treated with serially diluted concentrations of GSK (**b**) or ORY1001 (**c**) for 1 h (n=3).

d–f Flow cytometry analysis of LSD1 (**d**), PD-1 (**e**), and SLAMF6 (**f**) expression in *Cd4*^{Cre}*Lsd1*$^{f/f}$ (*Lsd1*^{cKO}) and *Lsd1*$^{f/f}$ $CD8^+$ T cells activated and expanded *in vitro* for 5 days with the treatment of GSK or Veh (n = 3). MFI, mean fluorescence intensity.

g–i Immunoblot of LSD1 (**g**) and flow cytometry analysis of PD-1 (**h**) and SLAMF6 (**i**) in *Rosa26*$^{Cre\text{-}ERT2}$*Lsd1*$^{f/f}$ (*Lsd1*^{iKO}) and *Lsd1*$^{f/f}$ $CD8^+$ T cells activated and expanded *in vitro* for 5 days with the treatment of GSK and/or 4-hydroxytamoxifen (4-OHT) as indicated (n = 3).

j, k Flow cytometry analysis of PD-1 (**j**) and SLAMF6 (**k**) expression in $CD8^+$ T cells treated with GSK, ORY1001, or Veh for 5 days (n = 3).

Data in this figure are presented as mean ± SD and are representative two independent experiments (**a**–**k**). Statistical significance was determined by two-sided unpaired t test (**e**, **f**, **h**–**k**). Source data are provided as a Source Data file.

Supplementary Figure 2

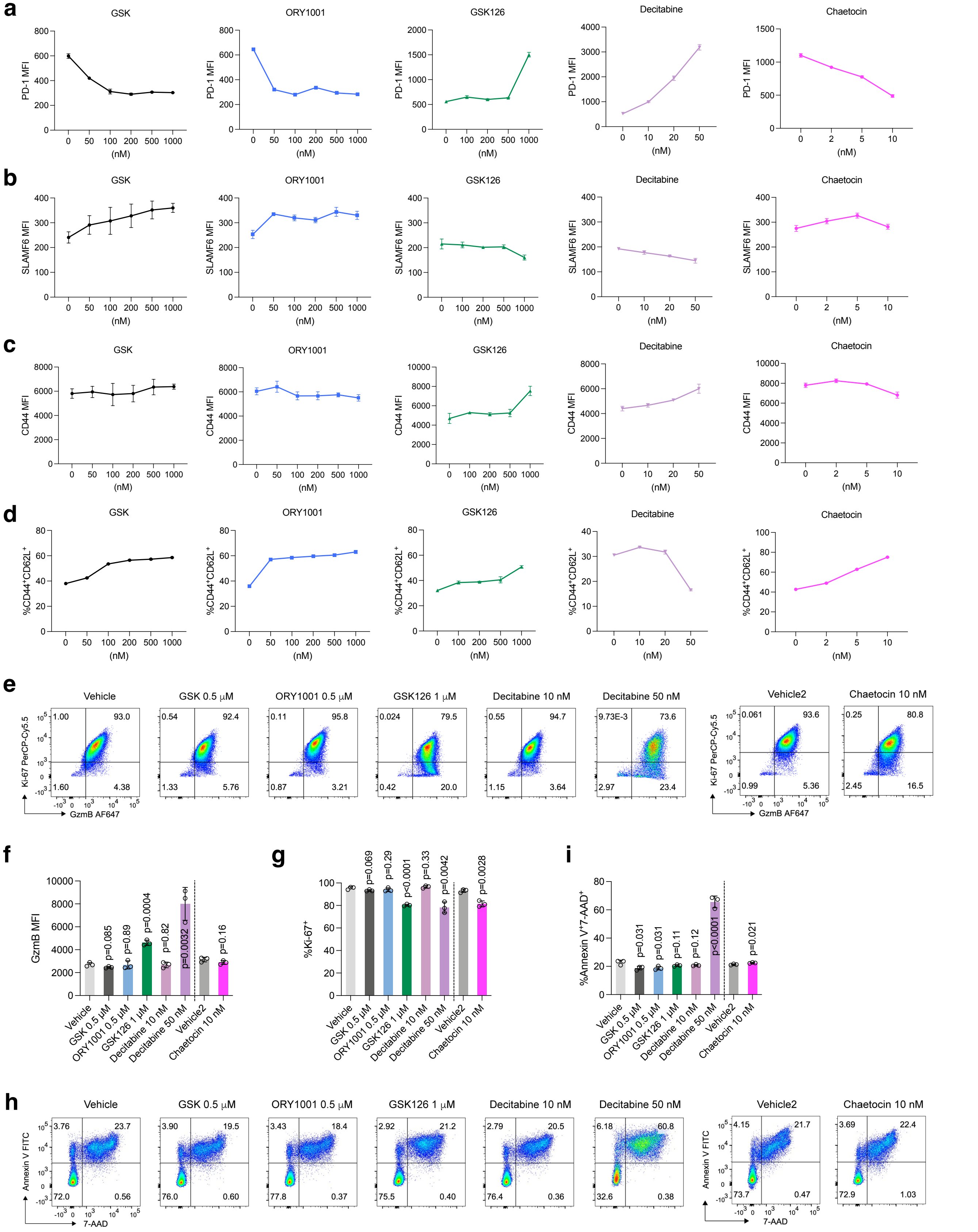

Supplementary Fig. 2 Effects of epigenetic modulators on T cell activation.

a–d Flow cytometry analysis of PD-1 (**a**), SLAMF6 (**b**), CD44 (**c**), and CD62L (**d**) expression in $CD8^+$ T cells activated and expanded *in vitro* for 5 days with the treatment of indicated epigenetic inhibitors (n = 3).

e–g Representative flow plots (**e**), GzmB expression (**f**), and percentages of Ki-67^+ cells (**g**) in $CD8^+$ T cells treated with indicated inhibitors (n = 3).

h, i Representative flow plots (**h**) and percentages of Annexin-V^+7-AAD^+ cells (**i**) in $CD8^+$ T cells treated with indicated inhibitors (n = 3).

Data in this figure are presented as mean ± SD and are representative of two independent experiments (**a**–**i**). Statistical significance was determined by two-sided unpaired t test (**f**, **g**, **i**) . Source data are provided as a Source Data file.

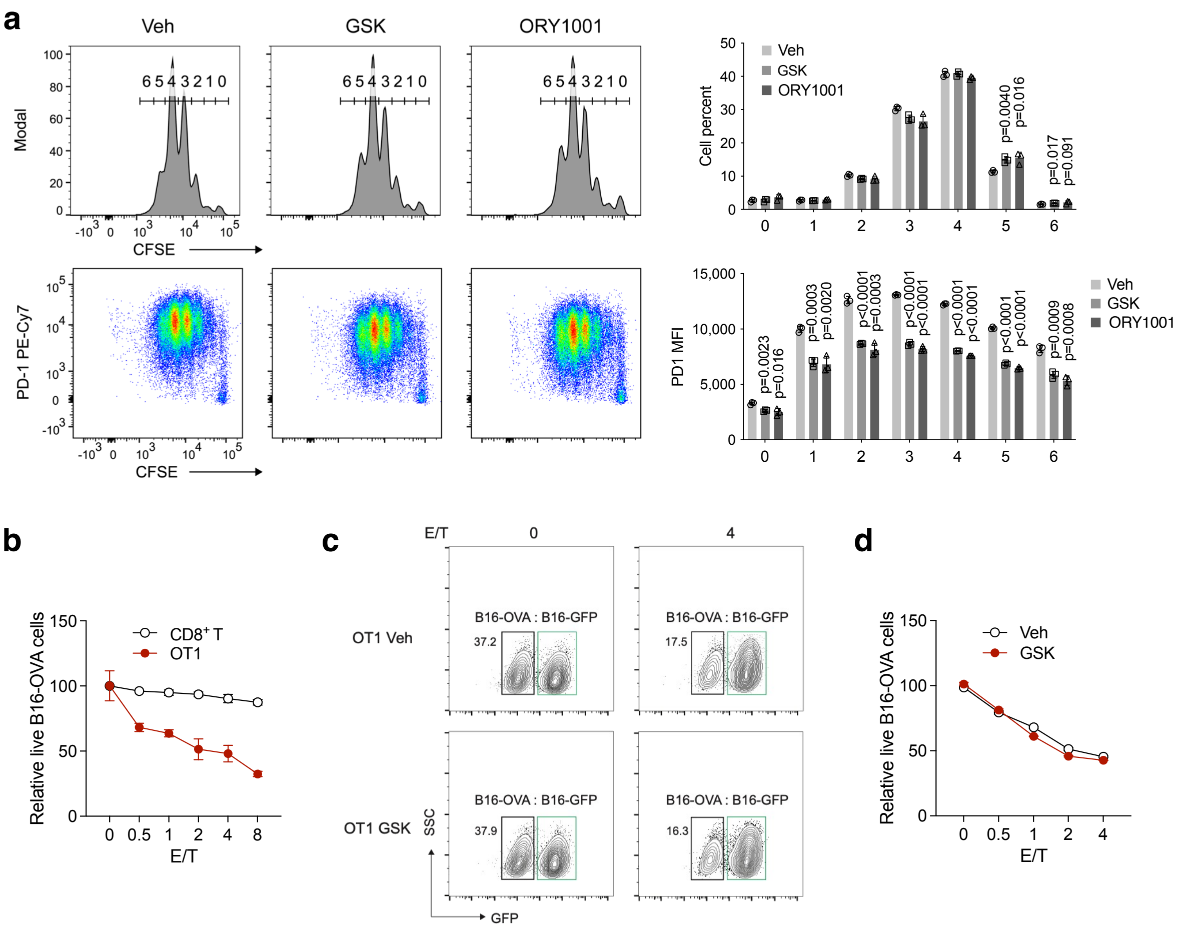

Supplementary Fig. 3 The cytolytic ability of effector OT1 cells is not affected by GSK treatment.

a Flow cytometry analysis of cell proliferation of CFSE-labeled $CD8^+$ T cells activated *in vitro* for 3 days with or without GSK treatment and PD-1 expression by $CD8^+$ T cells at different division numbers (n = 3).

b Antigen-dependent killing assay using *in vitro* activated and expanded OT1 or unmodified $CD8^+$ T cells as effectors and 1:1 mixed B16-OVA and B16-GFP cells as targets (n = 3). E/T, effector to target ratio.

c Representative flow plots showing the percentages of B16-OVA cells among live target cells after 18-h killing by GSK- or Veh-treated OT1 cells.

d Cytotoxicity of GSK- or Veh-treated OT1 cells after 5-day *in vitro* activation and expansion assessed by antigen-dependent killing assay (n = 3).

Data in this figure are presented as mean ± SD and are representative of two (**a**) or three (**b**–**d**) independent experiments. Statistical significance was determined by two-sided unpaired t test (**a**). Source data are provided as a Source Data file.

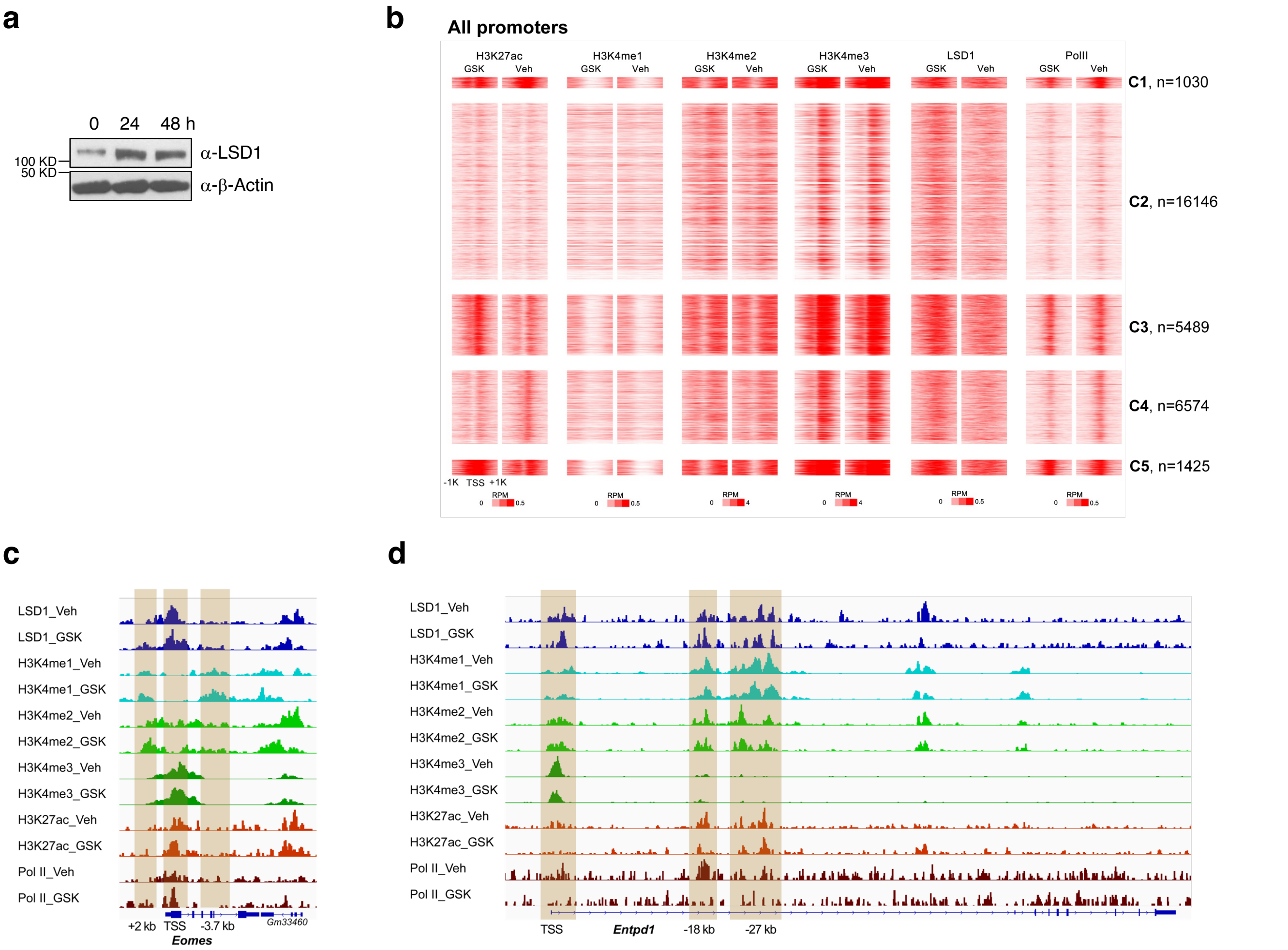

Supplementary Fig. 4 Epigenetic regulation of *Eomes* and *Entpd1* by LSD1 inhibition.

a Immunoblot of LSD1 in CD8⁺ T cells stimulated with anti-CD3/anti-CD28 for 24 or 48 h. Source data are provided as a Source Data file.

b Heatmaps of ChIP-seq signals at all promoters (TSS ± 1 kb) in GSK- or Veh-treated CD8⁺ T cells, generated by K-means clustering on H3K27ac signals.

c, d IGV snapshots showing ChIP-seq tracks at genomic loci of *Eomes* (**c**) and *Entpd1* (**d**).

The presented data are from one of two repeated experiments (**a**–**d**).

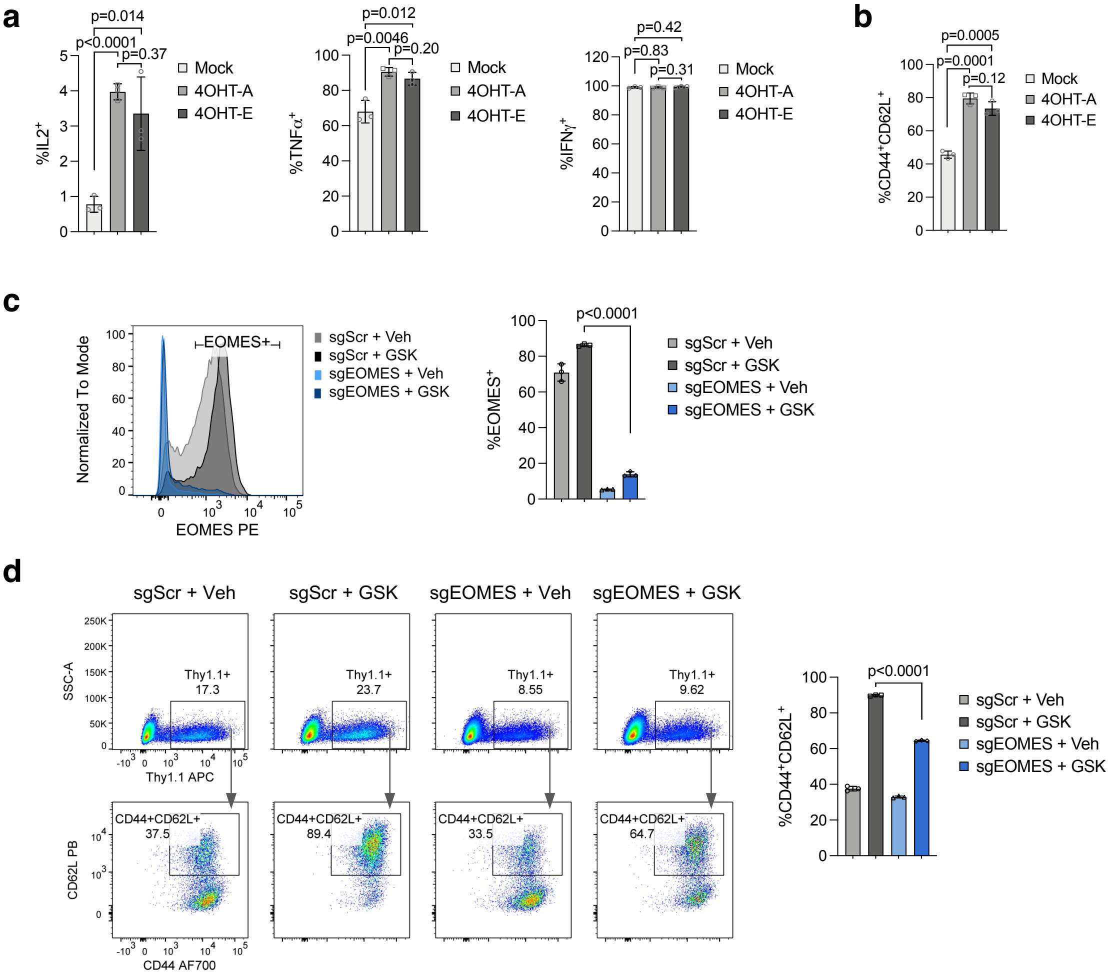

Supplementary Fig. 5 LSD1 inhibition promotes the central memory phenotype of CD8+ T cells in part through EOMES.

a, b Flow cytometry analysis of cytokine production (**a**) and percentages of CD44+CD62L+ cells (**b**) in *Rosa26*$^{Cre\text{-}ERT2}$*Lsd1*$^{f/f}$ CD8+ T cells treated with 4-OHT during the 2-day activation period (4OHT-A) or the 3-day expansion period (4OHT-E) (n = 3).

c, d Flow cytometry analysis of percentages of EOMES+ cells (**c**) and CD44+CD62L+ cells (**d**) in Rosa26-Cas9 CD8+ T cells transduced with sgScramble or sgEOMES and treated with GSK or Veh (n = 3).

Data in this figure are presented as mean ± SD and are representative of three independent experiments (**a**–**d**). Statistical significance was determined by two-sided unpaired t test (**a**–**d**). Source data are provided as a Source Data file.

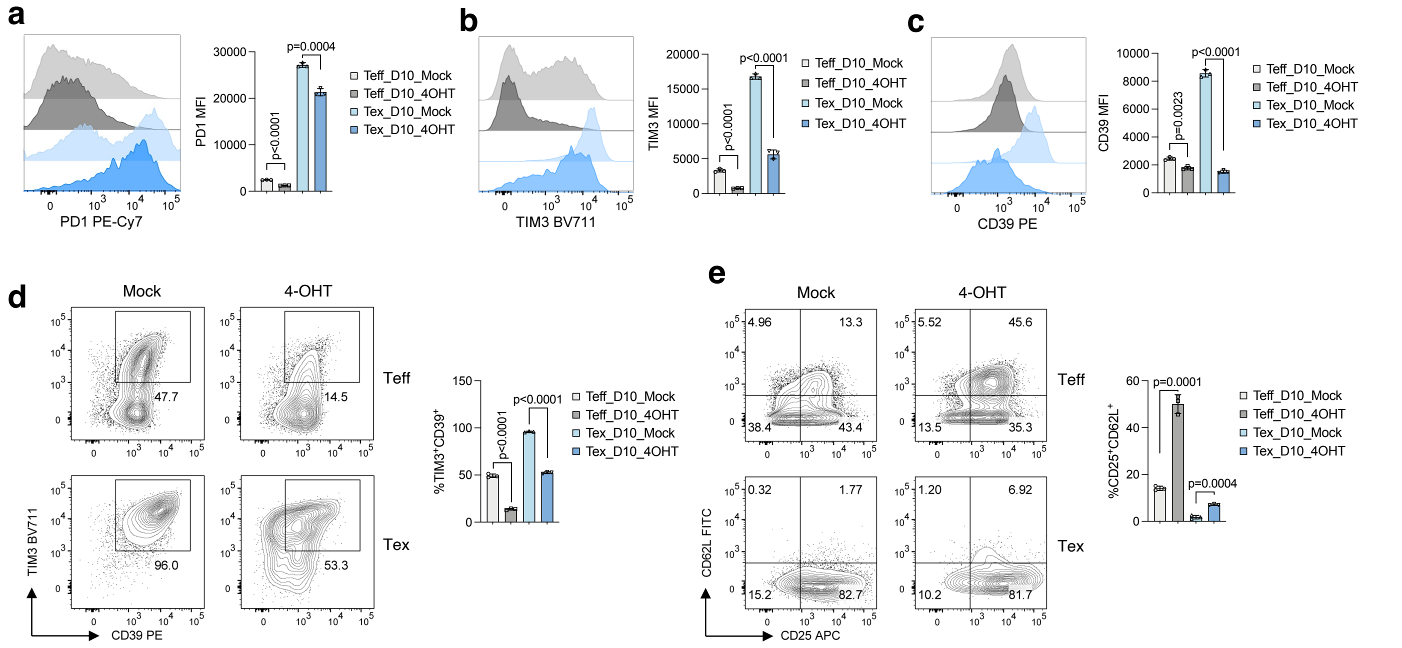

Supplementary Fig. 6 LSD1 ablation suppresses the *in vitro* induction of T cell exhaustion.

a–c Flow cytometry analysis of PD-1 (**a**), TIM-3 (**b**), and CD39 (**c**) expression in *Rosa26*$^{\textit{Cre-ERT2}}$*Lsd1*$^{f/f}$ CD8^{+} T cells after repeated stimulation with anti-CD3 (Tex) or continuous IL-2 expansion (Teff), with or without 4-OHT treatment during the initial TCR stimulation period (n = 3).

d, e Percentages of TIM-3^{+}CD39^{+} cells (**d**) and CD25^{+}CD62L^{+} cells (**e**) detected by flow cytometry (n = 3).

Data in this figure are presented as mean ± SD and are representative of two independent experiments (**a**–**e**). Statistical significance was determined by two-sided unpaired t test (**a–e**). Source data are provided as a Source Data file.

Supplementary Figure 7

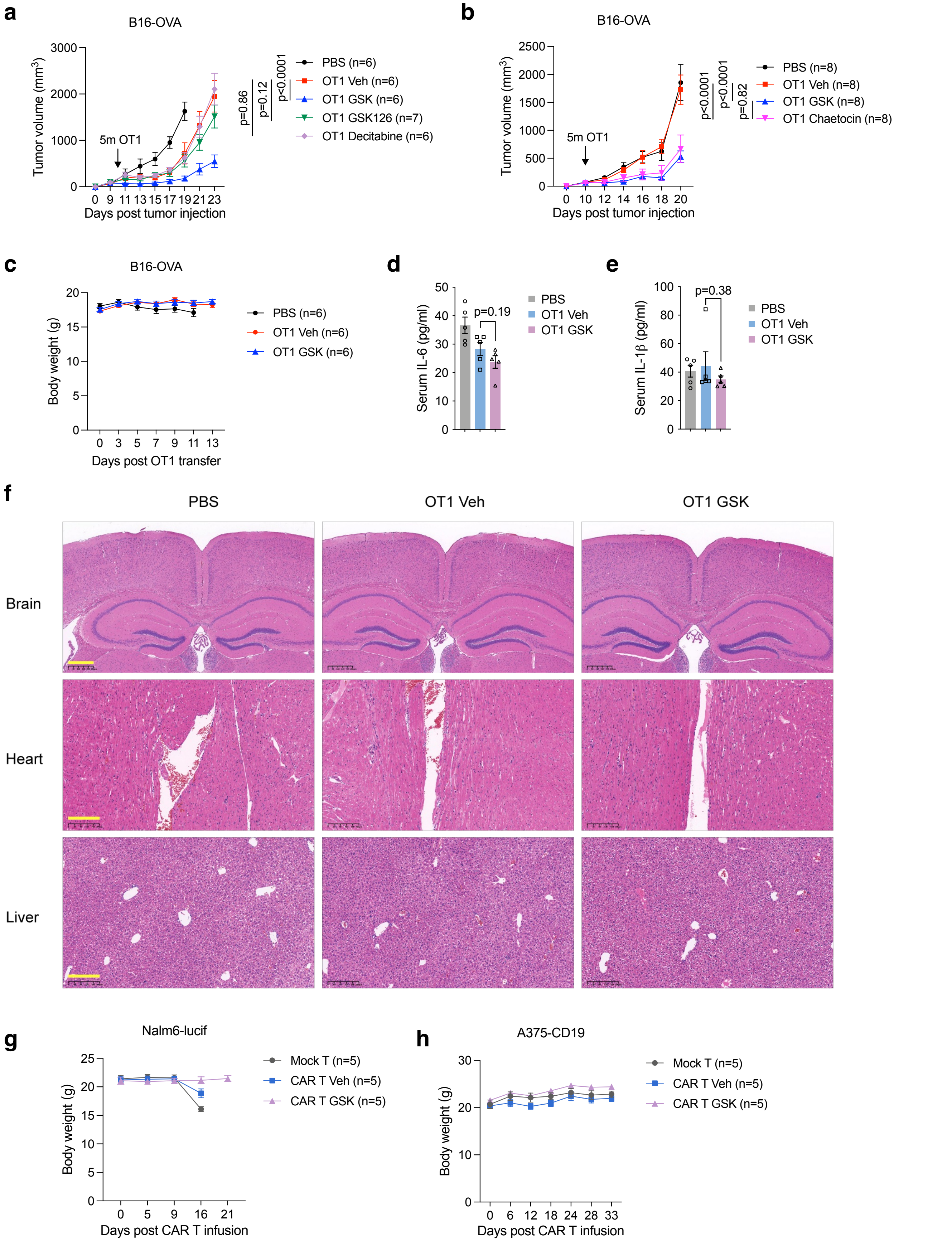

Supplementary Fig. 7 GSK Priming of T cells produces a superior antitumor effect without leading to toxicities.

a, b Tumor growth curves of mice subcutaneously inoculated with B16-OVA tumor cells and transferred with 5 million OT1 cells primed with different epigenetic inhibitors (n=6–8 mice per group as indicated).

c Body weights of B16-OVA tumor-bearing mice after transfer with 5 million GSK- or Veh-primed OT1 cells, or PBS (n=6 mice per group).

d, e ELISA quantification of serum IL-6 (**d**) and IL-1β (**e**) two weeks after OT1 cell transfer (n=5 mice per group).

f Representative histological sections of the brain, heart, and liver collected from recipient mice two weeks after OT1 cell transfer (n=3 mice per group). Scale bars, 400 µm for the brain and 200 µm for the heart and liver.

g, h Body weights of NCG mice carrying Nalm6-lucif leukemia (**g**) or A375-CD19 melanoma (**h**) after infusion of GSK- or Veh-primed CD19-CAR T cells, or mock T cells (n=5 mice per group).

Data are presented as mean ± SEM (**a**–**e**, **g**, **h**). Statistical significance was determined by two-way ANOVA (**a**, **b**) or two-sided unpaired t test (**d**, **e**). Source data are provided as a Source Data file.
