# Supplementary material for: Priming with LSD1 inhibitors promotes the persistence and antitumor effect of adoptively transferred T cells
Source: Nat Commun. 2024 May 21;15:4327. doi: 10.1038/s41467-024-48607-4 (PMC11109160; doi:10.1038/s41467-024-48607-4)
Supplement: Supplementary file 3 — Reporting Summary [file 41467_2024_48607_MOESM3_ESM.pdf]

nature portfolio

Corresponding author(s): Wanqiang Sheng, Jianya Zhou, Xiaowen Lyu

Last updated by author(s): Apr 28, 2024

Reporting Summary

Nature Portfolio wishes to improve the reproducibility of the work that we publish. This form provides structure for consistency and transparency in reporting. For further information on Nature Portfolio policies, see our Editorial Policies and the Editorial Policy Checklist.

Statistics

For all statistical analyses, confirm that the following items are present in the figure legend, table legend, main text, or Methods section.

n/a	Confirmed	
☐	☒	The exact sample size (n) for each experimental group/condition, given as a discrete number and unit of measurement
☐	☒	A statement on whether measurements were taken from distinct samples or whether the same sample was measured repeatedly
☐	☒	The statistical test(s) used AND whether they are one- or two-sided<br>*Only common tests should be described solely by name; describe more complex techniques in the Methods section.*
☒	☐	A description of all covariates tested
☐	☒	A description of any assumptions or corrections, such as tests of normality and adjustment for multiple comparisons
☐	☒	A full description of the statistical parameters including central tendency (e.g. means) or other basic estimates (e.g. regression coefficient) AND variation (e.g. standard deviation) or associated estimates of uncertainty (e.g. confidence intervals)
☐	☒	For null hypothesis testing, the test statistic (e.g. F, t, r) with confidence intervals, effect sizes, degrees of freedom and P value noted<br>*Give P values as exact values whenever suitable.*
☒	☐	For Bayesian analysis, information on the choice of priors and Markov chain Monte Carlo settings
☒	☐	For hierarchical and complex designs, identification of the appropriate level for tests and full reporting of outcomes
☒	☐	Estimates of effect sizes (e.g. Cohen's d, Pearson's r), indicating how they were calculated

Our web collection on statistics for biologists contains articles on many of the points above.

Software and code

Policy information about availability of computer code

Data collection	Flow data were acquired using FACSDiva 8.0.1 (BD Pharmingen). Bioluminescence imaging data were collected on a PhotonIMAGER Optima (Biospace Lab). RNA-seq data were collected on a Novaseq 6000 platform. ChIP-seq data were collected on a Novaseq 6000 platform and a DNBSEQ-T7 platform.
Data analysis	For RNA-seq analysis:<br>40 million raw reads for each sample were generated.<br>Raw reads in fastq format were first processed using Trimmomatic and the low-quality reads were removed to obtain the clean reads. Only clean reads passing the QC were used for subsequent analyses. The clean reads were mapped to the mouse reference genome (GRCm38) using hisat2 (version 2.1.0). FPKM of each gene was calculated using Cufflinks (version 2.2.1). The read counts of each gene were obtained by HTSeq-count (version 0.11.2). Differential expression analysis was performed using R package DESeq2 (version 1.22.2). To be called DEGs, fold change > 1.5 and q-value < 0.05 were set as the cutoff. The reported statistical significances were corrected for multiple testing using the Benjamini-Hochberg procedure with a false discovery rate less than 0.05. For gene ontology enrichment assessment, the differentially expressed genes were queried to the Gene Ontology Consortium using R package clusterProfiler (version 3.0.4) , with the specification of biological process.<br>For ChIP-seq analysis:<br>All reads were mapped to unique genomic regions of mm10 using Bowtie2. PCR duplicates were removed manually. Bedtools genome coverage function was used to generate bedgraph files for further analysis. To compare changes in ChIP-seq signals, libraries were normalized by random picking to obtain the same numbers of reads. Normalized reads were used to generate bedgraph files for comparison in IGV. MACS2 was used to call peaks using default parameters with p value cutoff at 0.001. Differential peaks were found using the edgeR R package at p < 0.05, with changes over 20% up/down.<br>Enhancers were defined by merging H3K4me1 and H3K4me2 peaks in both conditions but excluding H3K4me3 peaks and TSS ± 2.5kb.

Differential enhancers were found using the edgeR R package based on H3K27ac ChIP-seq signals with a p value cutoff at 0.05 and more than 3-fold changes in either condition. K-means clustering of enhancer heatmaps was done using Cluster3 on center ± 3 bins signals of H3K4me1 heatmaps, all heatmaps were plotted in this same order on center ± 1kb.
For deriving heatmaps of ChIP-seq signals, anchors plus flanking regions were binned equally to get a blank matrix (anchors × bins). To compare between samples, reads from the same antibody analysis were normalized by random pick. Normalized read pairs were mapped to each genomic bin with the bedtools intersect function to obtain read counts in each bin for the whole matrix. To normalize for sequencing depth, values in the matrix were divided by library sizes in millions to obtain reads per million per covered bin (RPMPCG or RPM), which was then visualized with Java TreeView to derive heatmaps. Average profiles of the ChIP-seq data were calculated and plotted using mean values of bins at the same distances from specific anchors. K-means clustering of ChIP-seq heatmaps was done using Cluster3 on center ± 3 bins signals of appropriate heatmaps. K-means clustering of promoter heatmaps was done using Cluster3 on TSSs ± 3 bins signals of H3K27ac or H3K4me2 heatmaps, all heatmaps were plotted in this same order on TSSs ± 1kb.
For flow cytometry: Data were analyzed using FlowJo (10.4).
For analysis of other data:
Statistical analyses were performed using GraphPad Prism (version 9.4) and statistical significance was determined by $p < 0.05$. A two-sided unpaired or paired Student's t-test was used for comparisons between the two groups. A two-way ANOVA test was used for multiple comparisons of tumor growth. A Log-rank (Mantel-Cox) test was used for comparing survival curves. Statistical data from experimental, biological, or technical replicates were presented as mean ± SEM or SD. The number of replicates and the statistical test used were indicated in the corresponding figure legends.

For manuscripts utilizing custom algorithms or software that are central to the research but not yet described in published literature, software must be made available to editors and reviewers. We strongly encourage code deposition in a community repository (e.g. GitHub). See the Nature Portfolio guidelines for submitting code & software for further information.

Data

Policy information about availability of data

All manuscripts must include a data availability statement. This statement should provide the following information, where applicable:

- Accession codes, unique identifiers, or web links for publicly available datasets
- A description of any restrictions on data availability
- For clinical datasets or third party data, please ensure that the statement adheres to our policy

The RNA-seq and ChIP-seq data generated in this study have been deposited in the Gene Expression Omnibus (GEO) database under accession codes: GSE248892 and GSE248891. The remaining data are available within the Article, Supplementary Information, or Source Data file. Source data are provided with this paper.

Research involving human participants, their data, or biological material

Policy information about studies with human participants or human data. See also policy information about sex, gender (identity/presentation), and sexual orientation and race, ethnicity and racism.

Reporting on sex and gender	Sex and gender were not considered in the experimental design.
Reporting on race, ethnicity, or other socially relevant groupings	Race, ethnicity, or other socially relevant groups were not considered in this study.
Population characteristics	Healthy adults
Recruitment	Healthy volunteers were recruited for donating 10 ml peripheral blood.
Ethics oversight	The protocol involves the use of human peripheral blood was approved Clinical Research Ethics Committee of the First Affiliated Hospital, College of Medicine, Zhejiang University (reference No. 2023-0349).

Note that full information on the approval of the study protocol must also be provided in the manuscript.

Field-specific reporting

Please select the one below that is the best fit for your research. If you are not sure, read the appropriate sections before making your selection.

☒ Life sciences ☐ Behavioural & social sciences ☐ Ecological, evolutionary & environmental sciences

For a reference copy of the document with all sections, see nature.com/documents/nr-reporting-summary-flat.pdf

Life sciences study design

All studies must disclose on these points even when the disclosure is negative.

Sample size	Group sizes for mouse experiments were determined empirically based on prior knowledge of the intra-group variation of tumor growth and drug treatment. We used different sample size for different tumor models and different types of experiments, based on previously published work (PMIDs: 33687985, 34819502). For in vitro experiments (T cell killing assay and flow cytometry analysis), sample sizes were determined empirically based on previous publications or protocols (PMIDs: 36002574, 34819502).
Data exclusions	No data were exclcuded from the analyses.

Replication	Replicates were used in experiments as noted in figure legends . Experiments presented for which replication was attempted were successfully replicated.
Randomization	Age and sex-matched mice were used for in vivo experiments. Mice were randomized prior to any treatment. For other in vitro randomization (killing assay, in vitro CD8+ T stimulation etc), samples were randomized for transfection or inhibitor treatment and no bias was introduced.
Blinding	For experiments with animals, mice were randomly assigned to separate cages for different treatments or injections. The investigators were not blinded when performing the experiments for cage labeling and staffing needs. Since a number of procedures (including cell injections, drug administrations, tumor measurements, tumor collections) were involved in the experiments, it is crucial to keep cage and animals properly labeled or tagged. It is not feasible to blind due to the complexity of the experiments and limited number of researchers. Even though blinding is technically impossible during many of the procedures, researchers were blinded during final step data collection and analysis. Each mouse was assigned an ID to ensure blinding and all analyses on the data was done in a blinded manner. The data acquiring process and analysis (including tumor measurements, flow cytometry analysis, etc) is a consistent and objective process and no personal bias is introduced.

Behavioural & social sciences study design

All studies must disclose on these points even when the disclosure is negative.

Study description	*Briefly describe the study type including whether data are quantitative, qualitative, or mixed-methods (e.g. qualitative cross-sectional, quantitative experimental, mixed-methods case study).*
Research sample	*State the research sample (e.g. Harvard university undergraduates, villagers in rural India) and provide relevant demographic information (e.g. age, sex) and indicate whether the sample is representative. Provide a rationale for the study sample chosen. For studies involving existing datasets, please describe the dataset and source.*
Sampling strategy	*Describe the sampling procedure (e.g. random, snowball, stratified, convenience). Describe the statistical methods that were used to predetermine sample size OR if no sample-size calculation was performed, describe how sample sizes were chosen and provide a rationale for why these sample sizes are sufficient. For qualitative data, please indicate whether data saturation was considered, and what criteria were used to decide that no further sampling was needed.*
Data collection	*Provide details about the data collection procedure, including the instruments or devices used to record the data (e.g. pen and paper, computer, eye tracker, video or audio equipment) whether anyone was present besides the participant(s) and the researcher, and whether the researcher was blind to experimental condition and/or the study hypothesis during data collection.*
Timing	*Indicate the start and stop dates of data collection. If there is a gap between collection periods, state the dates for each sample cohort.*
Data exclusions	*If no data were excluded from the analyses, state so OR if data were excluded, provide the exact number of exclusions and the rationale behind them, indicating whether exclusion criteria were pre-established.*
Non-participation	*State how many participants dropped out/declined participation and the reason(s) given OR provide response rate OR state that no participants dropped out/declined participation.*
Randomization	*If participants were not allocated into experimental groups, state so OR describe how participants were allocated to groups, and if allocation was not random, describe how covariates were controlled.*

Ecological, evolutionary & environmental sciences study design

All studies must disclose on these points even when the disclosure is negative.

Study description	*Briefly describe the study. For quantitative data include treatment factors and interactions, design structure (e.g. factorial, nested, hierarchical), nature and number of experimental units and replicates.*
Research sample	*Describe the research sample (e.g. a group of tagged Passer domesticus, all Stenocereus thurberi within Organ Pipe Cactus National Monument), and provide a rationale for the sample choice. When relevant, describe the organism taxa, source, sex, age range and any manipulations. State what population the sample is meant to represent when applicable. For studies involving existing datasets, describe the data and its source.*
Sampling strategy	*Note the sampling procedure. Describe the statistical methods that were used to predetermine sample size OR if no sample-size calculation was performed, describe how sample sizes were chosen and provide a rationale for why these sample sizes are sufficient.*
Data collection	*Describe the data collection procedure, including who recorded the data and how.*
Timing and spatial scale	*Indicate the start and stop dates of data collection, noting the frequency and periodicity of sampling and providing a rationale for these choices. If there is a gap between collection periods, state the dates for each sample cohort. Specify the spatial scale from which the data are taken*

Data exclusions	*If no data were excluded from the analyses, state so OR if data were excluded, describe the exclusions and the rationale behind them, indicating whether exclusion criteria were pre-established.*
Reproducibility	*Describe the measures taken to verify the reproducibility of experimental findings. For each experiment, note whether any attempts to repeat the experiment failed OR state that all attempts to repeat the experiment were successful.*
Randomization	*Describe how samples/organisms/participants were allocated into groups. If allocation was not random, describe how covariates were controlled. If this is not relevant to your study, explain why.*
Blinding	*Describe the extent of blinding used during data acquisition and analysis. If blinding was not possible, describe why OR explain why blinding was not relevant to your study.*

Did the study involve field work? ☐ Yes ☐ No

Field work, collection and transport

Field conditions	*Describe the study conditions for field work, providing relevant parameters (e.g. temperature, rainfall).*
Location	*State the location of the sampling or experiment, providing relevant parameters (e.g. latitude and longitude, elevation, water depth).*
Access & import/export	*Describe the efforts you have made to access habitats and to collect and import/export your samples in a responsible manner and in compliance with local, national and international laws, noting any permits that were obtained (give the name of the issuing authority, the date of issue, and any identifying information).*
Disturbance	*Describe any disturbance caused by the study and how it was minimized.*

Reporting for specific materials, systems and methods

We require information from authors about some types of materials, experimental systems and methods used in many studies. Here, indicate whether each material, system or method listed is relevant to your study. If you are not sure if a list item applies to your research, read the appropriate section before selecting a response.

Materials & experimental systems

n/a	Involved in the study	
☐	☒	Antibodies
☐	☒	Eukaryotic cell lines
☒	☐	Palaeontology and archaeology
☐	☒	Animals and other organisms
☒	☐	Clinical data
☒	☐	Dual use research of concern
☒	☐	Plants

Methods

n/a	Involved in the study	
☐	☒	ChIP-seq
☐	☒	Flow cytometry
☒	☐	MRI-based neuroimaging

Antibodies

Antibodies used	HRP-conjugated goat anti-rabbit IgG (Biosharp, BL003A); HRP-conjugated goat anti-mouse IgG (Biosharp, BL001A); PE-conjugated anti-CD8a (BioLegend, 300908); Zombie Aqua™ dye (BioLegend, 423101); CD8a BV605 (BioLegend, 100744); TCRβ BV510 (BioLegend, 109233); CD45.1 AF700 (Biolegend, 110724); CD45.2 PB (BioLegend, 109820); PD1 PE/Cyanine7 (BioLegend, 135215); TIM3 BV711 (BioLegend, 119727); TIM3 APC (BioLegend, 119705); CD39 PE (BioLegend, 143803); CD44 APC (BioLegend, 103012); CD62L FITC (BioLegend, 104405); CD62L Pacific blue (BioLegend, 161207); IL7Rα PE (BioLegend, 135009); KLRG1 FITC (BioLegend, 138409); CD25 APC (BioLegend, 102011); SLAMF6 APC (BioLegend, 134609); Ki67-PerCP/Cyanine5.5 (BD Pharmingen, 561284); Granzyme-B AF647 (BioLegend, 515405); LSD1 (Abcam, ab17721); IL2 FITC (BioLegend, 503805); TNFα FITC (BioLegend, 506303); IFNγ PE (BioLegend, 505807); EOMES PE (ThermoFisher Scientific, 12-4875-80); biotin-SP-AffiniPure F(ab)'2 fragment-specific goat anti-mouse IgG antibody (Jackson ImmunoResearch, 115-066-072); streptavidin-phycoerythrin (BioLegend, 405203); CD8a PE/Cyanine7 (BioLegend, 344711); PD1 PB (BioLegend, 329915); TIM3 FITC (BioLegend, 345021); CD39 PE (BioLegend, 328207); TNFα APC (BioLegend, 502913); IFNγ FITC (BioLegend, 502505); IL2 PE (BioLegend, 500306); anti-H3K4me1 (Abcam, ab8895); anti-H3K4me2 (EMD Millipore, 07-030); anti-H3K4me3 (Active Motif, 39060); anti-H3K27ac (Active Motif, 39034); anti-Pol II (Santa Cruz Biotechnology, sc-56767); rabbit normal IgG (Proteintech, 30000-0-AP); anti-Phospho-Stat5 (CST, 9359); anti-Phospho-PI3 Kinase p85 (CST, 4228); anti-Phospho-p44/42 MAPK (Erk1/2) (CST, 9101) Antibody; anti-p44/42 MAPK (Erk1/2) (CST, 4695).
Validation	Validation is present on the manufacturer's website. All the information about the validation data can be found using the catalogue number and supplier name shown as above. Our flow cytometry and immunoblot data are also consistent with the data shown on the manufacturer's websites.

Eukaryotic cell lines

Policy information about cell lines and Sex and Gender in Research

Cell line source(s)	Nalm6-lucif-EGFP cell line was a gift from Dongrui Wang. Raji cell line was a gift from Zhijian Cai. B16/F10 is a gift from David Fisher. B16-OVA is a gift from Zhuang Liu. A375 is a gift from Bin Zhao. Those cell lines were originally purchased from ATCC. HEK293T cell line were purchased from National Collection of Authenticated Cell Cultures.
Authentication	None of these cell lines were authenticated after being received.
Mycoplasma contamination	All cell lines were confirmed negative for mycoplasma using a PCR-based method.
Commonly misidentified lines (See ICLAC register)	No commonly misidentified cell lines were used.

Palaeontology and Archaeology

Specimen provenance	*Provide provenance information for specimens and describe permits that were obtained for the work (including the name of the issuing authority, the date of issue, and any identifying information). Permits should encompass collection and, where applicable, export.*
Specimen deposition	*Indicate where the specimens have been deposited to permit free access by other researchers.*
Dating methods	*If new dates are provided, describe how they were obtained (e.g. collection, storage, sample pretreatment and measurement), where they were obtained (i.e. lab name), the calibration program and the protocol for quality assurance OR state that no new dates are provided.*

☐ Tick this box to confirm that the raw and calibrated dates are available in the paper or in Supplementary Information.

Ethics oversight	*Identify the organization(s) that approved or provided guidance on the study protocol, OR state that no ethical approval or guidance was required and explain why not.*

Note that full information on the approval of the study protocol must also be provided in the manuscript.

Animals and other research organisms

Policy information about studies involving animals; ARRIVE guidelines recommended for reporting animal research, and Sex and Gender in Research

Laboratory animals	6~8-week-old female C57BL/6 mice were purchased from the Shanghai SLAC Laboratory Animal Co., Ltd. 6~7-week-old female NCG (NOD/ShiLtJGpt-Prkdcem26Cd52Il2rgem26Cd22/Gpt) mice (T001475) were purchased from GemPharmatech Co., Ltd. Lsd1flox/flox mice were generously provided by Dr. Weiguo Zou at Center for Excellence in Molecular Cell Science, CAS. Stat5flox/flox mice were generously provided by Xin-Yuan Fu at West China Hospital. OT1 (C57BL/6-Tg(TcraTcrb)1100Mjb/J, 003831), CD45.1 (B6.SJL-PtprcaPepcb/BoyJ, 002014), Cd4-Cre (Tg(Cd4-cre)1Cwi/BfluJ, 017336), and Rosa26-CreERT2 (B6.129-Gt(ROSA)26Sortm1(cre/ERT2)Tyj/J, 008463) mice were originally purchased from The Jackson Laboratory. Prior to all experiments, purchased mice were housed for one week to acclimate to the conditions at the Zhejiang University Laboratory Animal Center. All experimental mice were housed in specific pathogen-free conditions with a 12-h light/12-h dark cycle and controlled temperature (~22 °C). Animal experiments were performed in accordance with the animal care guidelines and with the prior approval by the Zhejiang University School of Medicine Institutional Animal Care and Use Committee.
Wild animals	No wild animals were involved.
Reporting on sex	Female mice were used as recipient mice of tumor implantation to optimize the engraftability.
Field-collected samples	Study did not involve field-collected samples.
Ethics oversight	All animal procedures were performed in accordance with animal care guidelines and with the prior approval by Zhejiang University School of Medicine Institutional Animal Care and Use Committee.

Note that full information on the approval of the study protocol must also be provided in the manuscript.

Clinical data

Policy information about clinical studies

All manuscripts should comply with the ICMJEguidelines for publication of clinical research and a completedCONSORT checklist must be included with all submissions.

Clinical trial registration	*Provide the trial registration number from ClinicalTrials.gov or an equivalent agency.*

Study protocol	*Note where the full trial protocol can be accessed OR if not available, explain why.*
Data collection	*Describe the settings and locales of data collection, noting the time periods of recruitment and data collection.*
Outcomes	*Describe how you pre-defined primary and secondary outcome measures and how you assessed these measures.*

Dual use research of concern

Policy information about [dual use research of concern]

Hazards

Could the accidental, deliberate or reckless misuse of agents or technologies generated in the work, or the application of information presented in the manuscript, pose a threat to:

No	Yes	
☐	☐	Public health
☐	☐	National security
☐	☐	Crops and/or livestock
☐	☐	Ecosystems
☐	☐	Any other significant area

Experiments of concern

Does the work involve any of these experiments of concern:

No	Yes	
☐	☐	Demonstrate how to render a vaccine ineffective
☐	☐	Confer resistance to therapeutically useful antibiotics or antiviral agents
☐	☐	Enhance the virulence of a pathogen or render a nonpathogen virulent
☐	☐	Increase transmissibility of a pathogen
☐	☐	Alter the host range of a pathogen
☐	☐	Enable evasion of diagnostic/detection modalities
☐	☐	Enable the weaponization of a biological agent or toxin
☐	☐	Any other potentially harmful combination of experiments and agents

Plants

Seed stocks	*Report on the source of all seed stocks or other plant material used. If applicable, state the seed stock centre and catalogue number. If plant specimens were collected from the field, describe the collection location, date and sampling procedures.*
Novel plant genotypes	*Describe the methods by which all novel plant genotypes were produced. This includes those generated by transgenic approaches, gene editing, chemical/radiation-based mutagenesis and hybridization. For transgenic lines, describe the transformation method, the number of independent lines analyzed and the generation upon which experiments were performed. For gene-edited lines, describe the editor used, the endogenous sequence targeted for editing, the targeting guide RNA sequence (if applicable) and how the editor was applied.*
Authentication	*Describe any authentication procedures for each seed stock used or novel genotype generated. Describe any experiments used to assess the effect of a mutation and, where applicable, how potential secondary effects (e.g. second site T-DNA insertions, mosiacism, off-target gene editing) were examined.*

ChIP-seq

Data deposition

☒ Confirm that both raw and final processed data have been deposited in a public database such as GEO.

☒ Confirm that you have deposited or provided access to graph files (e.g. BED files) for the called peaks.

Data access links<br>*May remain private before publication.*	ChIP-seq data generated in this study have been deposited in the Gene Expression Omnibus (GEO) database under accession code GSE248891.
Files in database submission	OT1_ChIP_GSK_H3K4me1_R1.fq.gz<br>OT1_ChIP_GSK_H3K4me1_R2.fq.gz

OT1_ChIP_GSK_H3K4me2_R1.fq.gz
OT1_ChIP_GSK_H3K4me2_R2.fq.gz
OT1_ChIP_GSK_Input1_R1.fq.gz
OT1_ChIP_GSK_Input1_R2.fq.gz
OT1_ChIP_GSK_LSD1_R1.fq.gz
OT1_ChIP_GSK_LSD1_R2.fq.gz
OT1_ChIP_veh_H3K4me1_R1.fq.gz
OT1_ChIP_veh_H3K4me1_R2.fq.gz
OT1_ChIP_veh_H3K4me2_R1.fq.gz
OT1_ChIP_veh_H3K4me2_R2.fq.gz
OT1_ChIP_veh_Input1_R1.fq.gz
OT1_ChIP_veh_Input1_R2.fq.gz
OT1_ChIP_veh_LSD1_R1.fq.gz
OT1_ChIP_veh_LSD1_R2.fq.gz
OT1_ChIP_GSK_Input2_R1.fq.gz
OT1_ChIP_GSK_Input2_R2.fq.gz
OT1_ChIP_GSK_H3K4me3_R1.fq.gz
OT1_ChIP_GSK_H3K4me3_R2.fq.gz
OT1_ChIP_GSK_H3K27ac_R1.fq.gz
OT1_ChIP_GSK_H3K27ac_R2.fq.gz
OT1_ChIP_GSK_PolII_R1.fq.gz
OT1_ChIP_GSK_PolII_R2.fq.gz
OT1_ChIP_veh_Input2_R1.fq.gz
OT1_ChIP_veh_Input2_R2.fq.gz
OT1_ChIP_veh_H3K4me3_R1.fq.gz
OT1_ChIP_veh_H3K4me3_R2.fq.gz
OT1_ChIP_veh_H3K27ac_R1.fq.gz
OT1_ChIP_veh_H3K27ac_R2.fq.gz
OT1_ChIP_veh_PolII_R1.fq.gz
OT1_ChIP_veh_PolII_R2.fq.gz
GSK_ChIP_H3K4me1_normbyinput.qnorm_100bp.bw
GSK_ChIP_H3K4me2_normbyinput.qnorm_100bp.bw
GSK_ChIP_H3K4me3_normbyinput.qnorm_100bp.bw
GSK_ChIP_H3K27ac_normbyinput.qnorm_100bp.bw
GSK_ChIP_LSD1_normbyinput.qnorm_100bp.bw
GSK_ChIP_polII_normbyinput.qnorm_100bp.bw
Veh_ChIP_H3K4me1_normbyinput.qnorm_100bp.bw
Veh_ChIP_H3K4me2_normbyinput.qnorm_100bp.bw
Veh_ChIP_H3K4me3_normbyinput.qnorm_100bp.bw
Veh_ChIP_H3K27ac_normbyinput.qnorm_100bp.bw
Veh_ChIP_LSD1_normbyinput.qnorm_100bp.bw
Veh_ChIP_polII_normbyinput.qnorm_100bp.bw
GSK_ChIP_H3K4me1_p0.001_peaks.broadPeak
GSK_ChIP_H3K4me2_p0.001_peaks.broadPeak
GSK_ChIP_H3K4me3_p0.001_peaks.broadPeak
GSK_ChIP_H3K27ac_p0.001_peaks.broadPeak
GSK_ChIP_LSD1_p0.001_peaks.narrowPeak
GSK_ChIP_polII_p0.001_peaks.narrowPeak
Veh_ChIP_H3K4me1_p0.001_peaks.broadPeak
Veh_ChIP_H3K4me2_p0.001_peaks.broadPeak
Veh_ChIP_H3K4me3_p0.001_peaks.broadPeak
Veh_ChIP_H3K27ac_p0.001_peaks.broadPeak
Veh_ChIP_LSD1_p0.001_peaks.narrowPeak
Veh_ChIP_polII_p0.001_peaks.narrowPeak

Genome browser session
(e.g. UCSC)

mm10

Methodology

Replicates

ChIP-seq experiments were repeated twice.

Sequencing depth

All replicates of ChIP-Seq are sequenced to a depth > 20 millions as paired-end reads.
GSK_H3K4me1_Brep1, Unique Pairs:34966852, Unique Unpaired:98759, Duplicate Pairs Optical:1707196, Duplicate Pairs Nonoptical:15726672, Duplicate Unpaired:156807, Unmapped:6018730
GSK_H3K4me2_Brep1, Unique Pairs:13435886, Unique Unpaired:35470, Duplicate Pairs Optical:678270, Duplicate Pairs Nonoptical:7104726, Duplicate Unpaired:64386, Unmapped:2842460
GSK_Input_Brep1, Unique Pairs:44848894, Unique Unpaired:75705, Duplicate Pairs Optical:2256964, Duplicate Pairs Nonoptical:29897842, Duplicate Unpaired:134053, Unmapped:424446
GSK_LSD1_Brep1, Unique Pairs:25490048, Unique Unpaired:50647, Duplicate Pairs Optical:1251350, Duplicate Pairs

	Nonoptical:14904616, Duplicate Unpaired:95003, Unmapped:17718144<br>Veh_H3K4me1_Brep1, Unique Pairs:26497938, Unique Unpaired:78932, Duplicate Pairs Optical:1245728, Duplicate Pairs Nonoptical:11029782, Duplicate Unpaired:101312, Unmapped:3762108<br>Veh_H3K4me2_Brep1, Unique Pairs:17510982, Unique Unpaired:46952, Duplicate Pairs Optical:914880, Duplicate Pairs Nonoptical:9171438, Duplicate Unpaired:78384, Unmapped:3711100<br>Veh_Input_Brep1, Unique Pairs:46068930, Unique Unpaired:83307, Duplicate Pairs Optical:1946232, Duplicate Pairs Nonoptical:25174566, Duplicate Unpaired:99828, Unmapped:368721<br>Veh_LSD1_Brep1, Unique Pairs:27767120, Unique Unpaired:43327, Duplicate Pairs Optical:1508050, Duplicate Pairs Nonoptical:16138646, Duplicate Unpaired:88774, Unmapped:16578041<br>GSK_H3K27ac_Brep1, Unique Pairs:24638112, Unique Unpaired:12232, Duplicate Pairs Optical:0, Duplicate Pairs Nonoptical:1774466, Duplicate Unpaired:2883, Unmapped:3649895<br>GSK_H3K4me3_Brep1, Unique Pairs:70188388, Unique Unpaired:15918, Duplicate Pairs Optical:0, Duplicate Pairs Nonoptical:3940040, Duplicate Unpaired:12942, Unmapped:3194404<br>GSK_Input_Brep2, Unique Pairs:26066400, Unique Unpaired:7623, Duplicate Pairs Optical:0, Duplicate Pairs Nonoptical:1549942, Duplicate Unpaired:1713, Unmapped:21922<br>GSK_polII_Brep1, Unique Pairs:63470974, Unique Unpaired:17808, Duplicate Pairs Optical:0, Duplicate Pairs Nonoptical:5937116, Duplicate Unpaired:5882, Unmapped:5598186<br>Veh_H3K27ac_Brep1, Unique Pairs:83498964, Unique Unpaired:13371, Duplicate Pairs Optical:0, Duplicate Pairs Nonoptical:8486964, Duplicate Unpaired:5657, Unmapped:6791084<br>Veh_H3K4me3_Brep1, Unique Pairs:67192624, Unique Unpaired:9644, Duplicate Pairs Optical:0, Duplicate Pairs Nonoptical:4194808, Duplicate Unpaired:8858, Unmapped:3641622<br>Veh_Input_Brep2, Unique Pairs:38947214, Unique Unpaired:5706, Duplicate Pairs Optical:0, Duplicate Pairs Nonoptical:3108288, Duplicate Unpaired:1923, Unmapped:22465<br>Veh_polII_Brep1, Unique Pairs:45459164, Unique Unpaired:9237, Duplicate Pairs Optical:0, Duplicate Pairs Nonoptical:4944522, Duplicate Unpaired:3709, Unmapped:6812580
Antibodies	anti-LSD1 (Abcam, ab17721), anti-H3K4me1 (Abcam, ab8895); anti-H3K4me2 (EMD Millipore, 07-030); anti-H3K4me3 (Active Motif, 39060); anti-H3K27ac (Active Motif, 39034); anti-Pol II (Santa Cruz Biotechnology, sc-56767); rabbit normal IgG (Proteintech, 30000-0-AP)
Peak calling parameters	All reads were mapped to unique genomic regions using Bowtie2 (v2.3.5.1) and the mm10 mus musculus genome release. PCR duplicates were removed manually by Samtools (v1.9). Bedtools (v2.27.1) genome Coverage function was used to derive bedgraph files for further analysis. To compare changes in ChIP-seq signals, libraries were normalized by random picking to obtain the same numbers of reads. MACS2 (v2.1.2) was used to call peaks using default parameters with Input data as control.
Data quality	Raw reads qualities were assessed by FASTQC prior to procession. Peaks were called by using input as controls (see methods for details) with default parameters in MACS2.
Software	MACS2.0 (Liu, 2014)https://github.com/taoliu/MACS<br>Bowtie2 (Langmead, 2012) https://bowtie-bio.sourceforge.net/bowtie2/index.shtml<br>HISAT2 (Kim, D et al., 2019) https://github.com/DaehwanKimLab/hisat2<br>StringTie (Pertea, M et al., 2015) https://ccb.jhu.edu/software/stringtie/<br>Samtools (Li et al., 2009) https://sourceforge.net/projects/samtools/files/<br>Picard Tools http://picard.sourceforge.net; https://broadinstitute.github.io/picard/<br>TOPHAT2 (Kim et al., 2013) https://github.com/infphilo/tophat<br>bedtools (Quinlan, 2014) http://bedtools.readthedocs.io/en/latest/<br>EdgeR （Robinson et al., 2010） https://bioconductor.org/packages/release/bioc/html/edgeR.html/<br>MAnorm (Shao et al., 2012) http://bcb.dfci.harvard.edu/~gcyuan/MAnorm/MAnorm.htm<br>Java treeview https://sourceforge.net/projects/jtreeview/files/<br>Cluster3 http://bonsai.hgc.jp/~mdehoon/software/cluster/software.htm<br>FIMO (Grant et al., 2011) http://meme-suite.org/<br>MEME (Bailey et al., 2006) http://meme-suite.org/

Flow Cytometry

Plots

Confirm that:

- [x] The axis labels state the marker and fluorochrome used (e.g. CD4-FITC).
- [x] The axis scales are clearly visible. Include numbers along axes only for bottom left plot of group (a 'group' is an analysis of identical markers).
- [x] All plots are contour plots with outliers or pseudocolor plots.
- [x] A numerical value for number of cells or percentage (with statistics) is provided.

Methodology

Sample preparation	Tumors were collected on day 5 or day 8 post adoptive T cell transfer and cut into 2 mm-sized pieces in RPMI1640 medium with the addition of type I collagenase (Worthington Biochemical Corporation, LS004194) and DNase I (Sigma-Aldrich, 10104159001). Tumor tissues were then digested at 37 °C for 20 ~ 30 min and passed through a 70 μm cell strainer to obtain

a single-cell suspension. To enrich leukocytes, samples were spun through a Percoll (GE Healthcare Life Sciences, 17-0891-01) gradient for 20 min at 2, 000 rpm without brake. Tumor-infiltrating leukocytes were collected from the interface of the 40% and 70% Percoll gradient, stained, and analyzed for fluorescent markers. In vitro cultured cells were collected by centrifugation and stained with fluorescent antibodies for flow cytometry analysis. 7-AAD (BioLegend, 420404) or Zombie NIR dye (BioLegend, 423106) was used to exclude dead cells.

Instrument: Stained single cell suspensions were run on a BD LSRFortessa.

Software: Flow data were acquired by FACSDIVA 8.0.1 (BD Pharmingen) and analyzed by FlowJo 10.4.1

Cell population abundance: Clearly divided into groups

Gating strategy: Use 7AAD or Zombie NIR to exclude dead cells, use CD45.1 or CD45.2, TCRb, and CD8a antibodies to identify CD8+ T cells, and then analyze the expression of other markers.

☐ Tick this box to confirm that a figure exemplifying the gating strategy is provided in the Supplementary Information.

Magnetic resonance imaging

Experimental design

Design type: *Indicate task or resting state; event-related or block design.*

Design specifications: *Specify the number of blocks, trials or experimental units per session and/or subject, and specify the length of each trial or block (if trials are blocked) and interval between trials.*

Behavioral performance measures: *State number and/or type of variables recorded (e.g. correct button press, response time) and what statistics were used to establish that the subjects were performing the task as expected (e.g. mean, range, and/or standard deviation across subjects).*

Acquisition

Imaging type(s): *Specify: functional, structural, diffusion, perfusion.*

Field strength: *Specify in Tesla*

Sequence & imaging parameters: *Specify the pulse sequence type (gradient echo, spin echo, etc.), imaging type (EPI, spiral, etc.), field of view, matrix size, slice thickness, orientation and TE/TR/flip angle.*

Area of acquisition: *State whether a whole brain scan was used OR define the area of acquisition, describing how the region was determined.*

Diffusion MRI ☐ Used ☐ Not used

Preprocessing

Preprocessing software: *Provide detail on software version and revision number and on specific parameters (model/functions, brain extraction, segmentation, smoothing kernel size, etc.).*

Normalization: *If data were normalized/standardized, describe the approach(es): specify linear or non-linear and define image types used for transformation OR indicate that data were not normalized and explain rationale for lack of normalization.*

Normalization template: *Describe the template used for normalization/transformation, specifying subject space or group standardized space (e.g. original Talairach, MNI305, ICBM152) OR indicate that the data were not normalized.*

Noise and artifact removal: *Describe your procedure(s) for artifact and structured noise removal, specifying motion parameters, tissue signals and physiological signals (heart rate, respiration).*

Volume censoring: *Define your software and/or method and criteria for volume censoring, and state the extent of such censoring.*

Statistical modeling & inference

Model type and settings: *Specify type (mass univariate, multivariate, RSA, predictive, etc.) and describe essential details of the model at the first and second levels (e.g. fixed, random or mixed effects; drift or auto-correlation).*

Effect(s) tested: *Define precise effect in terms of the task or stimulus conditions instead of psychological concepts and indicate whether ANOVA or factorial designs were used.*

Specify type of analysis: ☐ Whole brain ☐ ROI-based ☐ Both

Statistic type for inference

(See Eklund et al. 2016)

Specify voxel-wise or cluster-wise and report all relevant parameters for cluster-wise methods.

Correction

Describe the type of correction and how it is obtained for multiple comparisons (e.g. FWE, FDR, permutation or Monte Carlo).

Models & analysis

n/a	Involved in the study
☐	☐ Functional and/or effective connectivity
☐	☐ Graph analysis
☐	☐ Multivariate modeling or predictive analysis

Functional and/or effective connectivity

Report the measures of dependence used and the model details (e.g. Pearson correlation, partial correlation, mutual information).

Graph analysis

Report the dependent variable and connectivity measure, specifying weighted graph or binarized graph, subject- or group-level, and the global and/or node summaries used (e.g. clustering coefficient, efficiency, etc.).

Multivariate modeling and predictive analysis

Specify independent variables, features extraction and dimension reduction, model, training and evaluation metrics.
